# Plasma biomarkers of neurodegeneration in mild cognitive impairment with Lewy bodies

**DOI:** 10.1017/S0033291723001952

**Published:** 2023-12

**Authors:** Calum Alexander Hamilton, John O'Brien, Amanda Heslegrave, Rhiannon Laban, Paul Donaghy, Rory Durcan, Sarah Lawley, Nicola Barnett, Gemma Roberts, Michael Firbank, John-Paul Taylor, Henrik Zetterberg, Alan Thomas

**Affiliations:** 1 Translational and Clinical Research Institute, Newcastle University, Newcastle, UK; 2 Department of Psychiatry, University of Cambridge School of Clinical Medicine, Cambridge, UK; 3 UK Dementia Research Institute, London, UK; 4 Department of Neurodegenerative Disease, University College London, London, UK; 5 Nuclear Medicine Department, Newcastle upon Tyne Hospitals NHS Foundation Trust, Newcastle, UK; 6 Department of Psychiatry and Neurochemistry, University of Gothenburg, Gothenburg, Sweden; 7 Clinical Neurochemistry Laboratory, Sahlgrenska University Hospital, Gothenburg, Sweden; 8 Hong Kong Center for Neurodegenerative Diseases, Hong Kong, China; 9 Wisconsin Alzheimer's Disease Research Center, University of Wisconsin School of Medicine and Public Health, University of Wisconsin-Madison, Madison, WI, USA

**Keywords:** Alzheimer's disease, dementia with Lewy bodies, mild cognitive impairment, neurodegeneration, plasma biomarkers

## Abstract

**Background:**

Blood biomarkers of Alzheimer's disease (AD) may allow for the early detection of AD pathology in mild cognitive impairment (MCI) due to AD (MCI-AD) and as a co-pathology in MCI with Lewy bodies (MCI-LB). However not all cases of MCI-LB will feature AD pathology. Disease-general biomarkers of neurodegeneration, such as glial fibrillary acidic protein (GFAP) or neurofilament light (NfL), may therefore provide a useful supplement to AD biomarkers. We aimed to compare the relative utility of plasma A*β*42/40, *p*-tau181, GFAP and NfL in differentiating MCI-AD and MCI-LB from cognitively healthy older adults, and from one another.

**Methods:**

Plasma samples were analysed for 172 participants (31 healthy controls, 48 MCI-AD, 28 possible MCI-LB and 65 probable MCI-LB) at baseline, and a subset (*n* = 55) who provided repeated samples after ≥1 year. Samples were analysed with a Simoa 4-plex assay for A*β*42, A*β*40, GFAP and NfL, and incorporated previously-collected *p*-tau181 from this same cohort.

**Results:**

Probable MCI-LB had elevated GFAP (*p* < 0.001) and NfL (*p* = 0.012) relative to controls, but not significantly lower A*β*42/40 (*p* = 0.06). GFAP and *p*-tau181 were higher in MCI-AD than MCI-LB. GFAP discriminated all MCI subgroups, from controls (AUC of 0.75), but no plasma-based marker effectively differentiated MCI-AD from MCI-LB. NfL correlated with disease severity and increased with MCI progression over time (*p* = 0.011).

**Conclusion:**

Markers of AD and astrocytosis/neurodegeneration are elevated in MCI-LB. GFAP offered similar utility to *p*-tau181 in distinguishing MCI overall, and its subgroups, from healthy controls.

## Introduction

Alzheimer's disease (AD) and Lewy body disease are the two commonest neurodegenerative causes of cognitive impairment. These often overlap, with some degree of AD co-pathology being present in many cases of Lewy body disease at autopsy (McAleese et al., 2021). Antemortem, cases of dementia with Lewy bodies (DLB) are frequently positive for amyloid-*β* (A*β*) biomarkers, though less frequently tau positive (Ferreira et al., 2020).

Misdiagnosis is a particular concern early in the disease course, such as the prodromal ‘mild cognitive impairment’ (MCI) stage, when the full clinical picture may not yet be evident, and there is a greater likelihood of non-degenerative conditions being mistaken for early degenerative disease (e.g. depression-related cognitive dysfunctions, delirium, functional cognitive disorders, or misdiagnosed normal cognitive ageing). Recently developed plasma markers including AD specific and disease general biomarkers of astrocytosis and neuronal injury may help to address these diagnostic shortcomings (Zetterberg, 2019).

AD-specific markers may help to positively identify MCI due to AD (MCI-AD). There is a reduced ratio of amyloid-*β* (A*β*) 42 to A*β*40 in blood in both dementia due to AD and MCI-AD, and this reduction is associated with A*β* positivity on PET imaging (Nakamura et al., 2018). Concentrations of phosphorylated tau (*p*-tau) at threonine 181 (*p*-tau181), 217, or 231 are elevated in AD, including neuropathologically-confirmed cases and amyloid positive MCI-AD, relative to cognitively healthy individuals and many non-AD neurodegenerative diseases (Chong et al., 2021). However, A*β*42/40 is also reduced, and *p*-tau181 elevated, in autopsy-confirmed DLB with AD co-pathology (Smirnov et al., 2022). MCI with Lewy bodies (MCI-LB) also features elevated *p*-tau181 relative to controls which may likewise reflect the presence of AD (Thomas et al., 2022). However, not all cases of DLB feature AD co-pathology, though this is common (McAleese et al., 2021). AD-specific biomarkers such as A*β*42/40 or *p*-tau may therefore be less useful in screening for Lewy body disease.

In contrast to the aetiology-specific AD biomarkers, plasma biomarkers of neurodegeneration which differentiate several neurological diseases from normal ageing may help to confirm the presence of progressive neurological disease in an ambiguous cognitive syndrome. Glial fibrillary acidic protein (GFAP), a protein expressed by astrocytes and possible marker of astrocytosis/astrocytic activation (Liddelow & Barres, 2017), and neurofilament light chain (NfL), a marker of neuroaxonal injury (Karlsson, Rosengren, & Haglid, 1991), are elevated in several neurological diseases including, but not limited to AD and Lewy body disease (Gaetani et al., 2019; Schulz et al., 2021). While potentially less specific to AD-related change than A*β*42/40 or *p*-tau, disease-general markers of astrocytosis/neurodegeneration may have greater utility at detecting other aetiologies in addition to AD (Chouliaras et al., 2022).

We therefore aimed to assess whether MCI-LB featured a plasma biomarker profile of lowered A*β*42/40 ratio, increased phosphorylated tau, GFAP and NfL in comparison to healthy older adults, whether this differed from MCI-AD, and which markers best discriminated MCI and its subtypes from normal ageing. We hypothesised that:

1. GFAP and NfL would best differentiate all-cause MCI, and MCI-LB alone, from normal ageing.
2. A*β*42/40 ratio and *p*-tau181 would best differentiate MCI-AD from normal ageing.
3. A*β*42/40 ratio and *p*-tau181 would be lowered and elevated respectively in MCI-AD relative to MCI-LB, but would not differentiate these reliably due to the variable and unknown prevalence of AD pathology in people with MCI-LB.

We also aimed to explore any longitudinal changes in plasma biomarkers over time, hypothesising that these may progressively worsen due to underlying disease progression.

## Methods

### Participants

Participants were drawn from two longitudinal cohorts as previously described (Donaghy et al., 2018, 2022). Briefly, people aged ≥60 with a health service diagnosis of MCI were recruited from older person's memory, psychiatry, neurology and geriatric healthcare services in North East England. They were screened for inclusion, and retained if they did not have dementia, Parkinson's disease for >12 months prior to cognitive symptoms, a subjective-only cognitive impairment, or suspected frontotemporal or vascular aetiology.

Cognitively healthy comparators were recruited from friends and family of MCI participants, and through advertisement to local research involvement services.

All participants provided written, informed consent to participate.

### Clinical assessment, imaging, and diagnosis

All participants underwent detailed clinical, cognitive and imaging assessment at baseline. Clinical and cognitive assessments were repeated at longitudinal follow-ups (annually prior to Covid-19, with an adaptive schedule thereafter). A three-person expert panel of old age psychiatrists (PCD, JPT, AJT) independently reviewed clinical research notes from each visit and provided a consensus diagnosis of MCI or all-cause dementia for each patient (Albert et al., 2011; McKhann et al., 2011). The same panel also independently rated the presence or absence of four core clinical features of Lewy body disease: complex visual hallucinations, fluctuating cognition, parkinsonian motor features, and REM sleep behaviour disorder (McKeith et al., 2017).

At baseline, all participants were offered 123I-N-fluoropropyl-2*β*-carbomethoxy-3*β*-(4-iodophenyl) single-photon emission computed tomography (FP-CIT) dopaminergic imaging (first cohort) or FP-CIT and 123I-metaiodobenzylguanidine (MIBG) cardiac scintigraphy (second cohort). FP-CIT images were visually rated blind to clinical information as normal or abnormal by a panel of experienced imaging analysts (Roberts et al., 2021a). Cardiac denervation was quantified as the ratio of MIBG uptake of the heart relative to the mediastinum (HMR) (Roberts et al., 2021b) with an HMR abnormality cut-off < 1.85 based on assessment of our local healthy comparator sample (Kane et al., 2019).

Imaging and clinical characteristics were incorporated into clinical diagnosis to produce a differential classification of MCI as either MCI-AD (0 core clinical features of Lewy body disease, both biomarkers normal and fulfilling diagnostic criteria for MCI-AD) (Albert et al., 2011), possible MCI-LB (1 core clinical feature of Lewy body disease with no abnormal biomarker, or 0 core clinical features of Lewy body disease but 1 + abnormal biomarker) or probable MCI-LB (2 + core clinical features of Lewy body disease, or 1 core clinical feature and 1 + abnormal biomarker) (McKeith et al., 2020). These classifications were updated after each reassessment as new information came to light.

### Biomarker assessment

Blood samples were collected at study baseline and at repeated follow-up by venepuncture in EDTA tubes, plasma was isolated by centrifuging, aliquoted and frozen at −80 °C for storage. Samples were collected either at the same visit as cognitive assessment, or at medical review which occurred a median of 13 days later.

Plasma samples were analysed by the UK Dementia Research Institute Biomarker Laboratory using Single molecule array (Simoa) HD1 analyser and the Quanterix Simoa Human Neurology 4-Plex E for A*β*40, A*β*42, GFAP and NfL. All samples were analysed with the same batch of reagents, at the same time. Plasma *p*-tau181 concentration was previously measured by this laboratory and reported in this cohort (Thomas et al., 2022), and is included here for comparison.

The mean inter-assay % coefficients of variation (CV) were 3.80% for A*β*40, 3.17% for A*β*42, 6.13% for GFAP, and 4.24% for NfL.

### Statistical analysis

A*β*40 and A*β*42 concentrations were combined to a ratio of A*β*42/40 as in previous research (Zetterberg, 2019). Anticipating that GFAP and NfL concentrations would follow a log-normal distribution as previously described (Chouliaras et al., 2022), GFAP and NfL log concentrations were instead analysed. *p*-tau181 was likewise log transformed for analysis as previously described (Thomas et al., 2022).

Diagnostic group differences (controls *v.* probable MCI-LB, and MCI-AD *v.* probable MCI-LB) were examined with linear models as previously (Thomas et al., 2022), as were correlations between plasma markers and disease severity as assessed by the Addenbrooke's Cognitive Examination – Revised (ACE-R). In both cases, models were adjusted for age at baseline assessment. Longitudinal changes in biomarkers were assessed with linear or log-linear mixed models, including subject-level random intercept and correlated time slope, also adjusting for age at baseline. Correlations between plasma biomarkers were assessed with Pearson's correlation. Diagnostic classification analyses were undertaken using generalised linear models and the *pROC* package, with confidence intervals computed by DeLong's method.

Since possible MCI-LB is diagnostically uncertain, group comparisons did not include possible MCI-LB though raw data are included for these for further context, and they are included in all-cause MCI analyses.

All analyses were undertaken with *R* statistical software version 4.1.3. As an exploratory analysis, we did not adjust for multiple comparisons. Residuals of linear models were checked for normality with Q-Q plots.

### Ethics and data sharing

This study received favourable ethical approval from the NRES Committee North East – Newcastle and North Tyneside 2 (12/NE/0290 and 15/NE/0420). The authors assert that all procedures contributing to this work comply with the ethical standards of the relevant national and institutional committees on human experimentation and with the Helsinki Declaration of 1975, as revised in 2008.

Data from these studies are available upon request through Dementias platform UK.

## Results

Baseline characteristics of healthy controls and MCI sub-groups are presented in Table 1.

**Table 1.** Demographics and biomarker values at baseline for cognitively healthy older adults and MCI groups: Mean (s.d.) for continuous and Count (%) for categorical variables

Characteristic	Cognitively Healthy *N* = 31	MCI-AD *N* = 48	Poss. MCI-LB *N* = 28	Prob. MCI-LB *N* = 65
*Age* (*Years*)	74.2 (7.8)	76.7 (7.0)	73.4 (8.0)	75.5 (6.9)
*Female gender*	8 (26%)	30 (62%)	14 (50%)	16 (25%)
*Self-reported duration of cognitive symptoms* (*Years*)	–	3.0 (2.5)	3.4 (2.7)	3.7 (3.1)
*ACE-R total score*	92.9 (4.2)	81.7 (9.5)	77.4 (12.0)	80.4 (9.4)
*Aβ42/40 ratio*	0.068 (0.013)	0.064 (0.010)	0.061 (0.012)	0.063 (0.014)
*p-tau181 Concentration* (*pg/ml*)	1.65 (0.76)	2.85 (1.28)	2.47 (1.18)	2.33 (1.06)
*p-tau181 Not available*	7 (23%)	7 (15%)	5 (18%)	8 (12%)
*GFAP concentration* (*pg/ml*)	85.59 (50.73)	166.94 (81.58)	126.15 (56.96)	137.28 (81.57)
*NfL concentration* (*pg/ml*)	19.30 (9.07)	28.09 (12.80)	23.83 (9.67)	25.67 (15.08)

### Plasma markers

**Figure 1.**
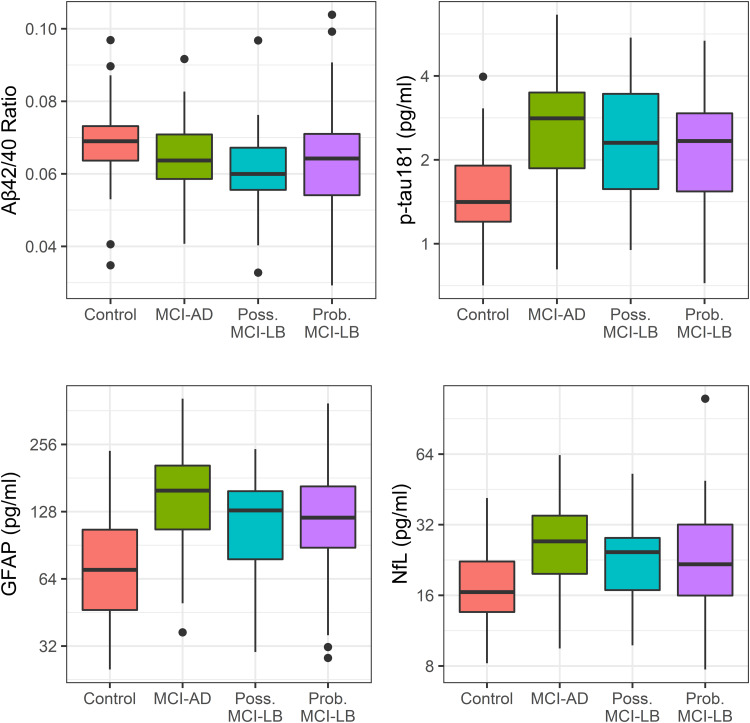
Baseline plasma markers of Alzheimer's disease (A*β*42/40 ratio and *p*-tau181) and neurodegeneration (GFAP and NfL) in each diagnostic group: quartile boxplot plus 1.5 × IQR whiskers.

Group differences in plasma biomarkers and summary statistics are shown in Table 1 and Fig. 1.

Plasma A*β*42/40 ratio did not significantly differ between probable MCI-LB and MCI-AD (Estimate = 0.001, [95% CI −0.003 to 0.005], *p* = 0.617) nor MCI-LB and healthy older adults (Estimate = 0.005 [−0.0002 to 0.0104], *p* = 0.063). MCI-AD did not have significantly lower A*β*42/40 ratio than controls (Estimate = −0.004 [−0.01 to 0.002], *p* = 0.170).

As previously reported, plasma *p*-tau181 was significantly elevated in probable MCI-LB relative to healthy older adults (Estimate = 0.31 [0.09 to 0.54], *p* = 0.007). *p*-tau181 was significantly higher in MCI-AD than both controls (Estimate = 0.52 [0.28 to 0.76], *p* < 0.001) and MCI-LB (Estimate = 0.21 [0.02 to 0.40], *p* = 0.033).

GFAP log concentration was significantly higher in probable MCI-LB cases than in healthy older adults (Estimate = 0.47 [0.22 to 0.71], *p* < 0.001). MCI-AD had significantly higher GFAP measures than both controls (Estimate = 0.71 [0.45 to 0.97], *p* < 0.001) and MCI-LB (Estimate = 0.24 [0.03 to 0.45], *p* = 0.028).

Log concentration of NfL in plasma was also significantly increased in probable MCI-LB relative to controls (Estimate = 0.25 [0.06 to 0.45], *p* = 0.012). MCI-AD also had significantly higher NfL than controls (Estimate = 0.36 [0.15 to 0.57], *p* = 0.001), but did not significantly differ from MCI-LB (Estimate = 0.11 [−0.06 to 0.28], *p* = 0.217).

We explored the possibility of differing ratios of ptau-181:NfL and ptau-181:GFAP, in both cases finding no significant differences between MCI-LB and MCI-AD (*p* = 0.382 and *p* = 0.522, respectively).

**Figure 2.**
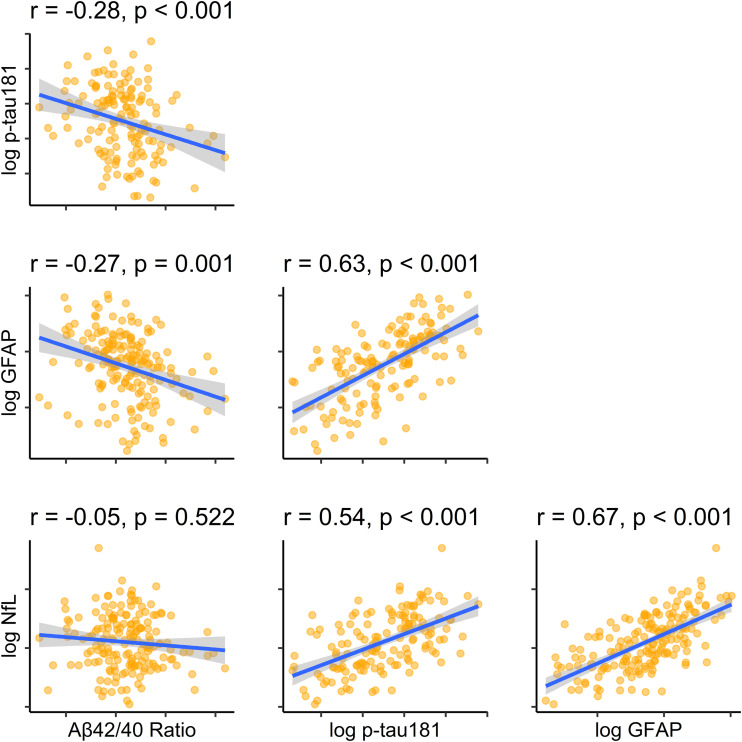
Bivariate correlations between plasma markers of Alzheimer's disease (A*β*42/40 ratio and *p*-tau181) and neurodegeneration (GFAP and NfL).

Pairwise associations between biomarkers were also assessed (see Fig. 2). A*β*42/40 was weakly negatively correlated with both plasma *p*-tau181 (*r* = −0.28, *p* < 0.001) and GFAP (*r* = −0.27, *p* = 0.001), but not significantly correlated with NfL (*r* = −0.05, *p* = 0.522). *p*-tau181 log concentration correlated strongly with GFAP log concentration (*r* = 0.63, *p* < 0.001), and moderately with NfL log concentration (*r* = 0.54, *p* < 0.001). Finally, GFAP and NfL log concentrations held a strong positive association (*r* = 0.67, *p* < 0.001).

### Association with baseline disease severity

Correlations between each plasma biomarker and baseline disease severity (total Addenbrooke's Cognitive Examination – Revised score; ACE-R) were examined with age-adjusted models within each MCI subgroup and overall.

In overall MCI, A*β*42/40 (*r* = 0.03, *p* = 0.755), GFAP (−0.12, *p* = 0.196), and *p*-tau181 (*r* = −0.07, *p* = 0.459) were not significantly associated with baseline age-adjusted ACE-R scores, while NfL held a weak negative association with baseline cognitive function (*r* = −0.24, *p* = 0.017).

Within MCI-AD A*β*42/40 (*r* = 0.04, *p* = 0.813) and GFAP (*r* = −0.23, *p* = 0.121) were not significantly associated with baseline ACE-R, but *p*-tau181 (*r* = −0.42 *p* = 0.007), and NfL (*r* = −0.48, *p* = 0.005) log concentrations held moderately significant correlations with greater disease severity.

Within probable MCI-LB, none of the plasma markers were significantly associated with baseline disease severity (see online Supplementary Fig. S1).

### Discriminative utility

**Figure 3.**
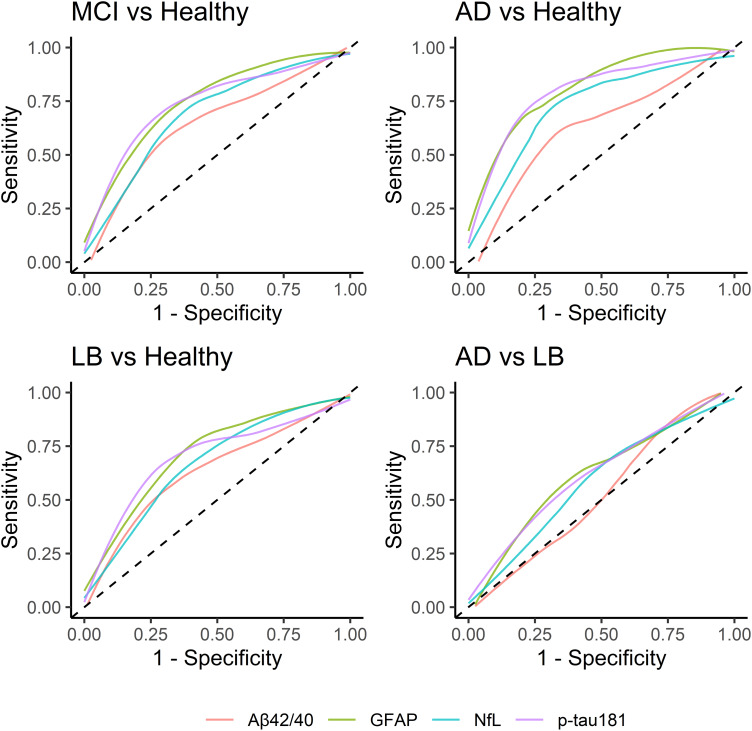
Discriminative utility of each plasma biomarker at differentiating MCI from healthy older adults, MCI-AD from healthy, MCI-LB from healthy, and MCI-AD from MCI-LB.

In discriminating any MCI case from healthy older adults, GFAP had the highest area under the ROC curve (AUC) with 0.75 [0.65 to 0.84], followed by *p*-tau181 (0.73 [0.63 to 0.84]), NfL (0.68[0.58 to 0.79]), and A*β*42/40 had the poorest discriminative utility (AUC of 0.64 [0.54 to 0.75]). GFAP was slightly superior to *p*-tau181 when sensitivity was favoured, and *p*-tau181 slightly superior when specificity was favoured (see Fig. 3).

Most biomarkers performed slightly better in discriminating MCI-AD from healthy controls (A*β*42/40 AUC = 0.63 [0.50 to 0.76], *p*-tau181 = 0.79 [0.68 to 0.91], GFAP = 0.82 [0.72 to 0.91], NfL = 0.72 [0.61 to 0.84]) and slightly worse in discriminating MCI-LB from controls (A*β*42/40 AUC = 0.63 [0.52 to 0.75], *p*-tau181 = 0.69 [0.57 to 0.82], GFAP = 0.71 [0.60 to 0.82], NfL = 0.67 [0.55 to 0.78]).

All plasma markers were generally poor at distinguishing MCI-AD from MCI-LB (A*β*42/40 AUC = 0.53 [0.42 to 0.64], *p*-tau181 = 0.62 [0.51 to 0.73], GFAP = 0.62 [0.51 to 0.72], NfL = 0.58 [0.47 to 0.69]).

### Longitudinal change in biomarkers

Fifty-five MCI participants had provided two or more plasma samples to permit an analysis of any change over time. Mean (s.d.) time between first and final sample collection were 1.4 years (0.50) for MCI-AD, 1.4 years (0.57) for possible MCI-LB and 1.7 years (0.61) for probable MCI-LB.

**Figure 4.**
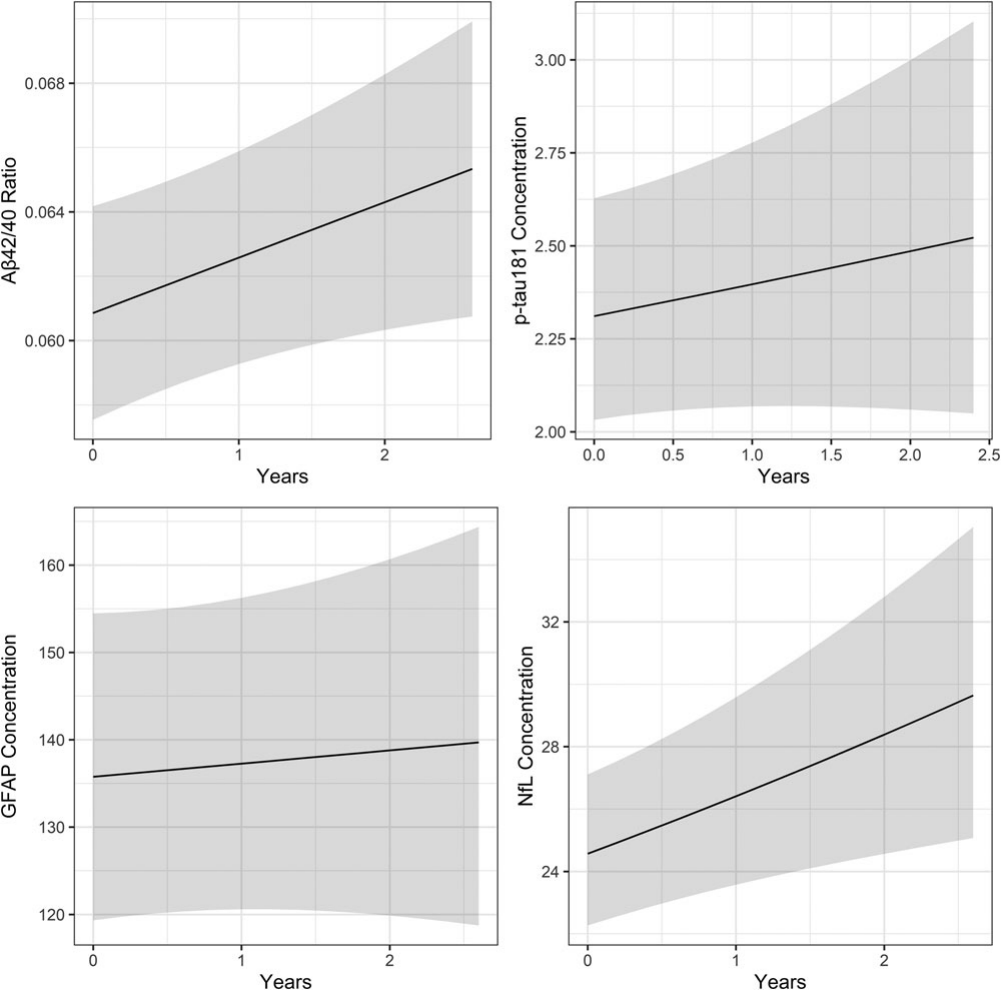
Significant increases in A*β*42/40 ratio, and NfL concentration over time in MCI.

In overall MCI, there was evidence of a marginally significant age-adjusted increase over time in A*β*42/40 (Estimate = +0.002 per year, *p* = 0.039) and NfL log-concentration (Estimate = +0.07 per year, *p* = 0.008) but not GFAP (Estimate = +0.01 per year, *p* = 0.675), or *p*-tau181 (Estimate = +0.04 per year, *p* = 0.305) (see Fig. 4).

## Discussion

We aimed to examine whether plasma biomarkers of AD (A*β*42/40 and *p*-tau181), astrocyte-expressed proteins (GFAP) and neurodegeneration (NfL) would differentiate MCI-LB from healthy controls, and MCI-AD from MCI-LB. We found evidence that both GFAP and NfL were elevated in MCI-LB, along with *p*-tau181 as previously described (Thomas et al., 2022). A*β*42/40 was not clearly decreased in MCI-LB or MCI-AD.

Of the assessed markers, plasma GFAP best differentiated all-cause MCI from healthy controls, followed by *p*-tau181, NfL, with A*β*42/40 having the poorest utility. This same pattern was replicated within disease-specific subgroups, including MCI-AD. All markers were poor at differentiating MCI-LB from MCI-AD.

We also aimed to examine whether there were any changes in biomarker levels over time. We found a slight normalisation of A*β*42/40 ratio over time in MCI cases, and a slight increase in NfL over time.

These results suggest that in both MCI overall, and in disease-specific subgroups MCI-AD and MCI-LB, GFAP and *p*-tau181 differentiate between MCI cases and non-cases with similar accuracy. While AD-specific biomarkers correlate with amyloid and tau deposition (Chong et al., 2021), these may not necessarily reflect consequential pathology in all cases and so may be relatively elevated in individuals with unrecognised asymptomatic AD within the healthy comparator group, reducing the specificity in differentiating MCI from healthy controls. Conversely, individuals without AD pathology may experience cognitive symptoms due to other processes (e.g. Lewy body disease or cerebrovascular disease), reducing the sensitivity to identify MCI.

GFAP, a disease-general marker of neurodegeneration, effectively differentiated both MCI-LB and MCI-AD from controls. This is consistent with previous research at the dementia stage which demonstrated that GFAP is elevated in both AD and Lewy body disease, though not in progressive supranuclear palsy or frontotemporal dementias (Chouliaras et al., 2022), with the exception of progranulin-associated cases (Heller et al., 2020); the latter aetiologies were excluded from these cohorts, and so these results may not translate to all MCI subtypes beyond MCI-AD and MCI-LB.

While NfL was less accurate than GFAP in distinguishing MCI from controls, this was moderately associated with age-adjusted disease severity in MCI. There was also evidence that levels of NfL in plasma were increasing over the course of MCI, suggesting that this may become more useful later in the disease course. GFAP, by contrast, remained stable. This may be consistent with the greater utility of NfL in distinguishing Lewy body disease from controls at the dementia stage (Chouliaras et al., 2022), though this requires further exploration and validation.

GFAP and NfL were strongly correlated, consistent with previous research findings (Elahi et al., 2020), though possibly asynchronous, with NfL still increasing at this stage: previous research has also supported that NfL, but not GFAP, progressively increases over the course of MCI and dementia due to AD (Simrén et al., 2021). Both measures were associated with *p*-tau181, but weakly correlated at best with A*β*42/40, suggesting that the former may have a closer association with astrocytic and neuronal dysfunction than the latter in MCI. These correlations are consistent with those seen in previous MCI and AD cohorts (Palmqvist et al., 2023).

There was an unexpected increase (i.e. normalisation) of A*β*42/40 over time in MCI, which is inconsistent with the expected worsening of underlying A*β* pathology. While previous data have been ambiguous as to whether A*β*42/40 ratio lowers over time in MCI (Simrén et al., 2021), given the small effect size and marginal significance of this result we are not confident that this represents a genuine normalisation of A*β*42/40 over the course of MCI. This may instead reflect a plateauing effect of the underlying pathology at the prodromal/clinical transition.

None of the plasma biomarkers appeared to be useful in distinguishing MCI-AD from MCI-LB. This is consistent with the recognised prevalence of AD co-pathology in DLB at autopsy (McAleese et al., 2021), and supports that this may also be present early in MCI-LB. Whether this co-pathology is sufficient to affect the clinical manifestation or disease progression at this stage is less clear: we have previously demonstrated that *p*-tau181 concentration is associated with disease progression in MCI-AD, but not MCI-LB (Thomas et al., 2022). In contrast, when dementia is clinically manifest, lower A*β*42/40 is associated with disease progression in DLB (Chouliaras et al., 2022).

These findings highlight the need for sensitive markers of alpha-synuclein pathology; while available biomarkers such as FP-CIT and MIBG are highly specific to DLB and MCI-LB, the sensitivity is lower than at the dementia stage (Roberts et al., 2021a, 2021b). We also demonstrate here that AD and neurodegeneration plasma biomarkers do not reliably differentiate MCI-AD from MCI-LB. Synuclein assays have been recently shown to detect prodromal and clinically manifest Lewy body disease in cerebrospinal fluid (Iranzo et al., 2021), skin punch samples (Mammana et al., 2021), and recently in blood (Kluge et al., 2022): disease-specific synuclein assays may therefore offer a method of reliably differentiating MCI-LB from MCI-AD, when more clinically accessible (i.e. blood-based) biomarkers are available.

## Strengths and limitations

This study benefitted from the inclusion of a clinically well-characterised cohort, with several years of follow-up, and differentially classified as MCI-AD or MCI-LB using current consensus criteria. However, as participants were recruited at the early stages of disease, autopsy validation of clinical diagnoses are not available for the majority of individuals, though follow-up and approach for brain tissue donation are ongoing.

Similarly, we did not have confirmatory CSF or PET testing available for A*β* or tau. Previous work has typically examined the utility of these biomarkers to differentiate A*β*-positive cases from A*β*-negative cases of MCI or dementia, and so we are unable to directly compare our AUC values to these (Benedet et al., 2021; Chouliaras et al., 2022; Palmqvist et al., 2023).

We present confidence intervals and *p* values unadjusted for multiple comparisons, and so encourage caution in interpreting these. While several reported estimates would be robust to any such adjustment, other estimates have a high degree of uncertainty: confidence intervals around the plasma biomarker AUCs are wide, and so these require further replication. Fewer participants had longitudinal biomarker measurements available, limiting the capability to assess disease-specific trajectories meaningfully.

While we have previously shown both MCI groups in these cohorts to reliably decline in cognition (Hamilton et al., 2021b, 2021c), and convert to dementia (Hamilton et al., 2021a), it is possible that an unknown number of these MCI cases may have a non-degenerative aetiology, such as a functional cognitive disorder mimicking MCI-AD or MCI-LB (Ball et al., 2020). Such unrecognised cases might not be expected to feature either an AD-like or broadly neurodegenerative biomarker profile, and so may lower the accuracy of these plasma biomarkers in this setting. Future research may benefit from assessing the relationship between these biomarkers and the longitudinal course of disease progression to assess whether the use of these might improve the identification of a progressive neurodegenerative MCI.

## Conclusions

GFAP assays performed comparably to *p*-tau181 in differentiating MCI from healthy controls, both overall and within MCI-AD or MCI-LB specifically. Markers of neurodegeneration or astrocytic protein expression may therefore help identify symptomatic neuropathological changes in AD and other diseases. Plasma AD and neurodegenerative markers had limited utility in differentiating MCI-AD from MCI-LB, highlighting a need for synucleinopathy-specific biomarkers.

## Supporting information

Hamilton et al. supplementary material Hamilton et al. supplementary material 1

Hamilton et al. supplementary material Hamilton et al. supplementary material 2

## Supplementary material

The supplementary material for this article can be found at https://doi.org/10.1017/S0033291723001952.
