# Supplementary figures and images for: Plasma biomarkers of neurodegeneration in mild cognitive impairment with Lewy bodies
Source: Psychol Med. 2023 Jul 25;53(16):7865–73. doi: 10.1017/S0033291723001952 (PMC10755229; doi:10.1017/S0033291723001952)

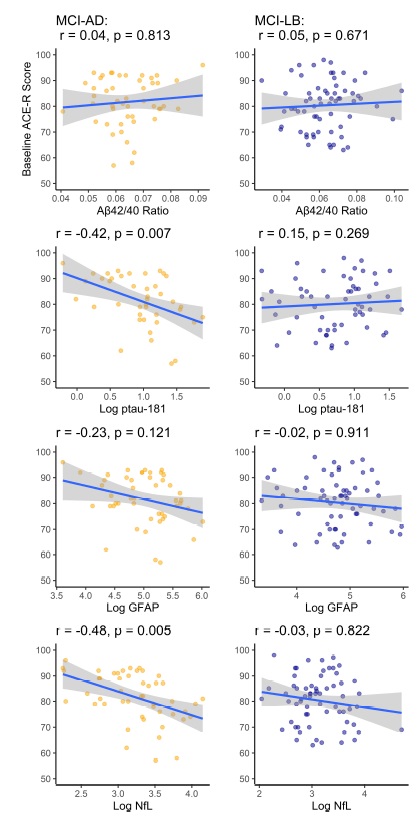

Supplement: Hamilton et al. supplementary material — 1 [file S0033291723001952sup001.jpg]
