# Supplementary material for: Plasma biomarkers of neurodegeneration in mild cognitive impairment with Lewy bodies
Source: Psychol Med. 2023 Jul 25;53(16):7865–73. doi: 10.1017/S0033291723001952 (PMC10755229; doi:10.1017/S0033291723001952)
Supplement: Hamilton et al. supplementary material — 2 [file S0033291723001952sup002.docx]

**Supplementary Table S1. Baseline demographics of subsample with longitudinal biomarker values available: Mean (SD) for continuous and Count (%) for categorical variables.**

| **Characteristic** | **Cognitively Healthy**  **N = 5** | **MCI-AD N = 20** | **Poss. MCI-LB**  **N = 9** | **Prob. MCI-LB**  **N = 23** |
| --- | --- | --- | --- | --- |
| *Age (Years)* | 76.0 (5.4) | 79.0 (6.8) | 69.8 (7.2) | 75.3 (6.9) |
| *Female Gender* | 1 (20%) | 13 (65%) | 4 (44%) | 5 (22%) |
| *Addenbrooke's Cognitive Examination - Revised* | 95.0 (2.3) | 80.5 (10.0) | 80.9 (12.9) | 82.1 (8.0) |
| *Self-Reported Duration of Cognitive Symptoms (Years)* | - | 3.6 (3.3) | 2.4 (1.8) | 3.9 (3.3) |
|  | | | | |
